# The prognostic role of extended preoperative hypercoagulability work-up in high-risk microsurgical free flaps: a single-center retrospective case series of patients with heterozygotic factor V Leiden thrombophilia

**DOI:** 10.1186/s12893-022-01639-3

**Published:** 2022-05-14

**Authors:** Florian Falkner, Benjamin Thomas, Martin Aman, Eva-Maria Risse, Gerhard Wittenberg, Emre Gazyakan, Leila Harhaus, Amir K. Bigdeli, Ulrich Kneser, Christian A. Radu

**Affiliations:** 1 Department of Hand, Plastic and Reconstructive Surgery, Burn Center, BG Trauma Center Ludwigshafen, Plastic- and Hand Surgery, University of Heidelberg, Ludwig-Guttmann-Str. 13, 67071 Ludwigshafen, Germany; 2 Department of Anesthesiology, Intensive Care Medicine and Pain Management, BG Trauma Clinic Ludwigshafen, Ludwigshafen, Germany

**Keywords:** Factor V Leiden, Activated protein C resistance, Free flap reconstruction, Thrombosis, Free flap failure, Flap anticoagulation

## Abstract

**Introduction:**

Hypercoagulability is associated with an increased risk of microvascular complications and free flap failures. The authors present their experience and approach to diagnosing and treating patients with heterozygotic factor V Leiden (hFVL) thrombophilia undergoing free flap reconstruction.

**Methods:**

Between November 2009 and June 2018, 23 free flap surgeries were performed in 15 hypercoagulable patients with hFVL. According to the timing of perioperative hypercoagulability work-up, they were grouped into flaps with established diagnoses prior to surgery (Group A) versus flaps with unknown diagnoses prior to surgery (Group B). Baseline characteristics and perioperative complications were compared between both groups, including revision surgeries due to microvascular thromboses, acute bleedings, hematomas, flap necroses, and reconstructive failures.

**Results:**

HFVL mutations had been confirmed preoperatively in 14 free flap surgeries (61%, Group A), whereas in 9 free flap surgeries (39%, Group B), mutations were only diagnosed postoperatively after the occurrence of microvascular thromboses had warranted extended hypercoagulability work-up. The overall rate of intraoperative flap thromboses was 9% (n = 2), whereas the overall rate of postoperative flap thromboses was 43% (n = 10). The corresponding salvage rates were 100% (n = 2/2) for intraoperative and 40% (n = 4/10) for postoperative pedicle thromboses. A total of five free flaps were lost (22%). Upon comparison, flaps with an unconfirmed diagnosis prior to surgery were at ten times higher risk for developing total necroses (flaps lost in Group B = 4/9 versus Group A = 1/14; OR: 10.4; 95% CI 1.0, 134.7; p = 0.03).

**Conclusion:**

Meticulous preoperative work-up of patients with any history of hypercoagulability can help reduce free flap loss rates, thus improving surgical outcomes and increasing patient safety.

## Introduction

Despite the continuous progress in microsurgery and increasing expertise in postoperative monitoring for early signs of flap malperfusion, flap loss still occurs in some patients, mainly because of microvascular pedicle thromboses. This may occur due to technical errors (e.g. backwall stitches, pedicle kinking, or intima lesions), or local influences, such as inflammation and scarring [1–3]. However, patients with previously undetected hypercoagulability also pose a significant challenge in microsurgery, particularly because most free flap reconstructions are undertaken without preoperative testing thereof [4].

In this context, factor V Leiden (FVL) mutation is an important factor predisposing patients to an increased risk of intra- and postoperative microvascular thromboses [5, 6]. The prevalence of FVL in Europe is reported to amount up to 15% [7]. In patients carrying the FVL mutation, factor V cannot be adequately inactivated. By way of background, activated protein C (APC) cleaves the coagulation factors Va and VIIIa and thus regulates thrombin production [8]. An impaired cleaving activity of APC leads to prolonged activation of factor Va and VIIIa [8]. This phenomenon of activated protein C (APC) resistance (APC-R) predisposes to venous thrombosis in particular [9]. Up to 95% of APC-R are caused by a point mutation in position 1691 in the factor V gene in chromosome 1 (G1691A) [10]. This substitution results in a change of arginine (A) to glutamine (G) at the cleavage site of protein C [10, 11], ultimately resulting in what is known as FVL [11, 12]. In heterozygotes (hFVL), the risk for thrombosis is 5 to 10 times higher when compared to the general population [12]. Preoperative anamnesis, basic and extended laboratory testing all play important roles for detecting hypercoagulability [4, 13]. However, experience with these high-risk patients in the setting of microsurgery is still scarce. Therefore, this article aims at elaborating on our experience with hypercoagulable patients carrying hFVL mutations undergoing microsurgical free flap reconstructions, emphasizing the importance and prognostic role of timely perioperative work-up and subsequent anticoagulatory treatment.

## Patients and methods

The study has been performed in accordance with the guidelines and regulations of the Declaration of Helsinki and has been approved by the responsible ethics committee of the Rhineland-Palatinate Chamber of Physicians (Mainz, Germany, IRB approval reference number: 2021-15883). Our department’s medical records from November 2009 through June 2018 were screened for cases of intra- or postoperative flap pedicle thromboses or patients that underwent thrombophilia screenings in the context of microsurgical free flap reconstructions. Chart review included patient demographic data, prevalent conditions, such as diabetes mellitus or coronary heart disease as well as specific individual risk factors, such as previous thrombotic events (deep vein thrombosis [DVT] or pulmonary artery embolism [PAE]), abortions, or positive family history for thrombosis. In addition, preoperative hematologic testing results, permanent anticoagulatory medication, peri- and postoperative medication, including any anticoagulation therapy, were analyzed. Intraoperative surgical complications, such as the occurrences of any flap pedicle thromboses, were catalogued. Postoperative surgical complications, which required additional surgery, were considered as “major” complications. The primary endpoint studied were the incidence of total flap losses, secondary endpoints included re-explorations due to acute vascular complications, such as thrombosis, acute bleeding, or hematoma, as well as partial flap necroses greater than 5% of the flap, and reconstructive failures, defined as any necroses requiring repeated flap surgery, amputation, or palliative wound care.

### Perioperative thrombophilia assessment and respective case versus control grouping

**Fig. 1:**
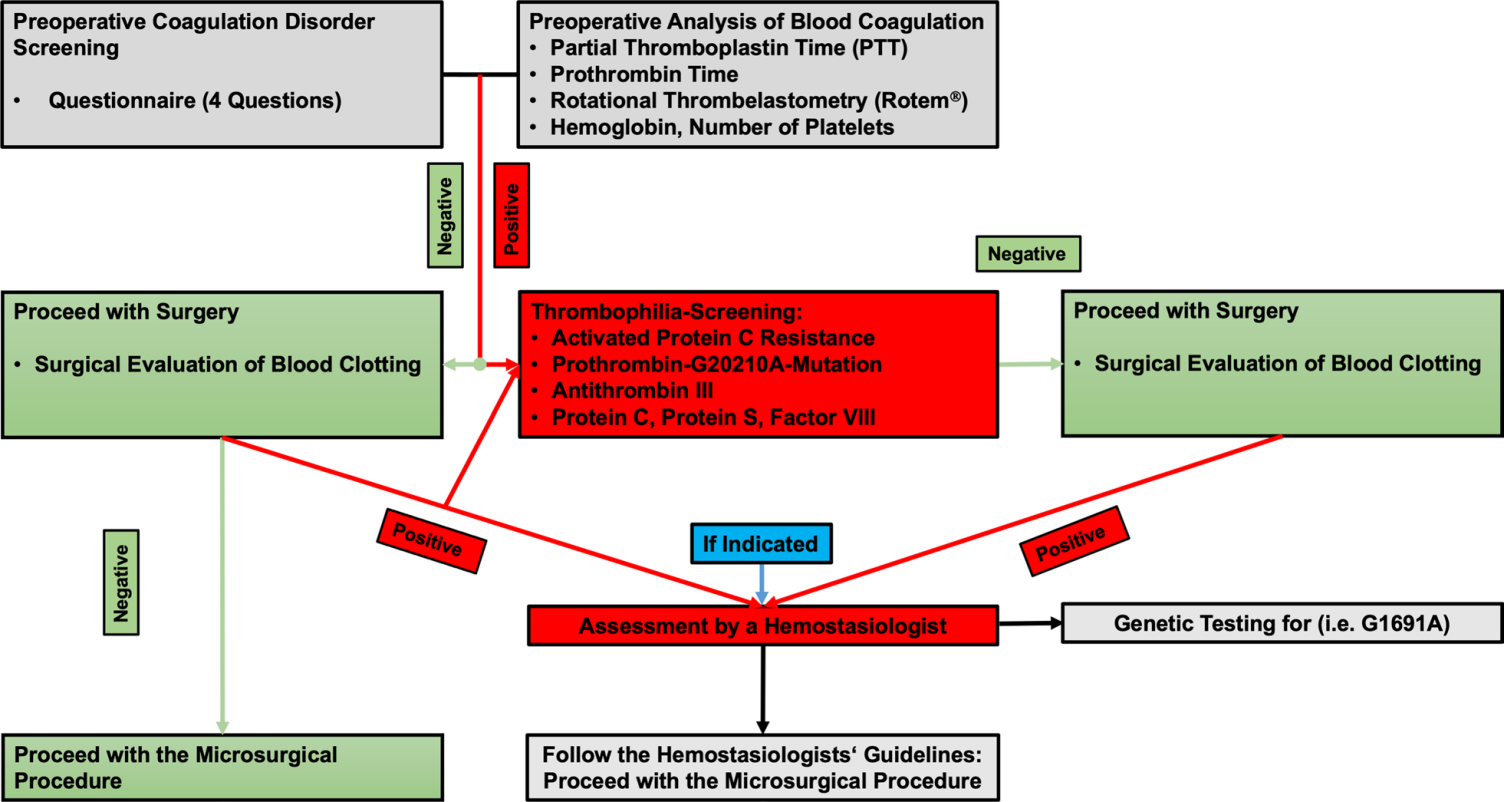
Algorithm for preoperative microsurgical risk assessment

Prior to free flap surgery, all patients are asked to answer a standardized mandatory questionnaire aimed at detecting (1) any personal or family history of DVT or PAE; (2) any past miscarriages; or (3) any personal or (4) family history of previously diagnosed coagulation disorders. In addition, blood samples were analyzed for hemoglobin, platelet count, conventional coagulation-tests, antithrombin III activity, fibrinogen levels, activated partial thromboplastin time (aPTT), prothrombin time. If hypercoagulability was suspected, patients were immediately referred to a hemostaseologist for further testing. In patients with one or more positive questionnaire items, the following thrombophilia screening was carried out in order to detect hereditary or acquired coagulation disorders: testing for APC-R, prothrombin-G20210A-mutation, antithrombin III, protein C, protein S, and factor VIII. Thrombophilia screening for APC-R was performed using the Coatest APC^®^ Resistance (Haemochrom Diagnostica GmbH, Essen, Germany). Subsequently, genetic testing for factor V-Leiden mutation (G1691A) confirmed the diagnosis. Figure 1 illustrates the routine preoperative work-up at our department. According to the timing of the extended genetic perioperative hypercoagulability work-up, all identified surgeries were grouped into flaps with preoperatively established diagnoses (Group A) versus flaps with postoperatively established diagnoses (Group B).

### Anticoagulatory treatment for patients with confirmed hFVL mutation

**Fig. 2:**
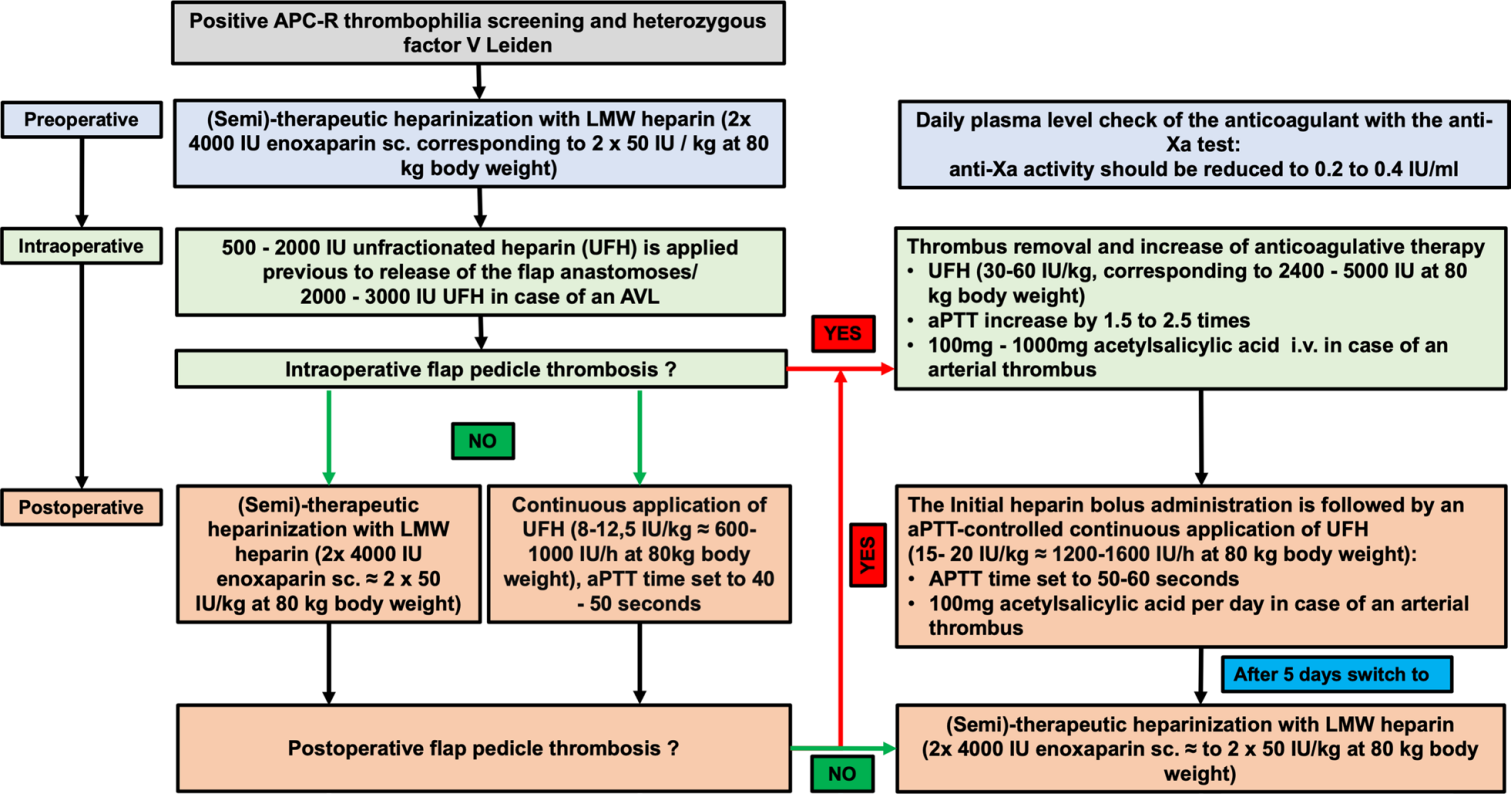
Anticoagulatory regimen in flaps with confirmed hFVL

For patients with confirmed hFVL mutation, a “semi-therapeutic” heparinization with low molecular weight heparin (LMWH, 2 × 4000 IU enoxaparin sc. corresponding to 2 × 50 IU/kg at 80 kg body weight) was administered preoperatively with daily anti-Xa activity monitoring (targeted at 0.2 to 0.4 IU/ml). Intraoperatively, 500–2000 IU (international units) of unfractioned heparin (UFH) were applied prior to releasing the flap anastomosis. Following peri- and postoperative pedicle thromboses, the anticoagulant therapy was immediately increased by means of UFH at 30–60 IU/kg, corresponding to 2400–5000 IU, in addition to emergency free flap re-exploration, thrombus removal, and re-anastomosis. Thus, the aPTT was increased 1.5- to 2.5-fold. After arterial thromboses, 100–1000 mg acetylsalicylic acid were administered intravenously, and 100 mg of oral acetylsalicylic acid were continued for at least 4 weeks. UFH administration was continued at 600–1000 IU/h, corresponding to 8–12.5 IU/kg at 80 kg body weight, with a target aPTT of 50–60 s. After 5 days, anticoagulatory treatment was switched back to LMWH at a semi-therapeutic dose, if no further thrombotic event occurred. Figure 2 shows the anticoagulatory treatment guideline used at our department.

### Statistical analysis

Statistical data analysis was performed using GraphPad Prism 8.0 (GraphPad Software, Inc., San Diego, CA). Data are presented as means with standard deviations (SD) or proportions with percentages. The Chi-squared test was used to assess the group differences in flap necrosis incidences and the odds ratio (OR) was derived with the corresponding 95% confidence interval (CI). An error probability of p < 0.05 was considered statistically significant.

## Results

**Fig. 3:**
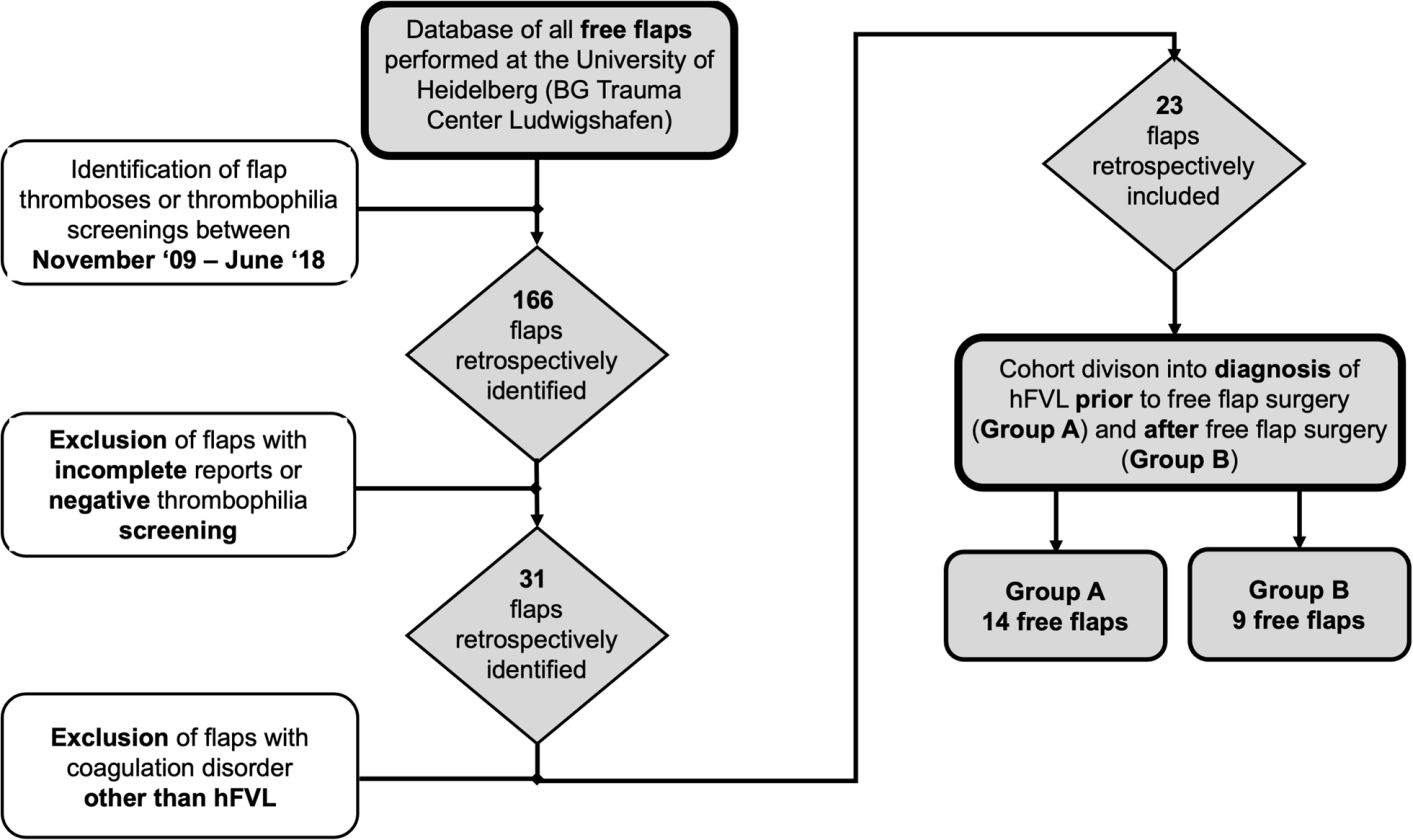
Flowchart of retrospective patient inclusion and exclusion criteria

Between November 2009 and June 2018, a total of 166 flap pedicle thromboses and perioperative thrombophilia work-ups were identified. After exclusion of flaps with incomplete reports or negative thrombophilia screenings, 31 flaps with thrombophilia remained. Of these, 23 flaps were performed in 15 patients with hypercoagulability due to heterozygotic FVL mutation (G1691A). The most frequent co-mutation was Prothrombin (PT) gene mutation (n = 15; 65%). Furthermore, three patients with homozygous FVL were identified in the study period and excluded in accordance with the exclusion criteria. Figure 3 illustrates the inclusion and exclusion criteria for retrospective analysis. Indications for free flap surgery in this cohort were trauma (n = 12), oncological defects (n = 7) and wound infections (n = 4). Free flap reconstructions were performed with deep inferior epigastric perforator flaps (n = 7), latissimus dorsi flaps (n = 7), anterolateral thigh perforator flaps (n = 3), rectus abdominis muscle flaps (n = 2), gracilis muscle flaps (n = 2), one parascapular and one combined latissimus dorsi and parascapular flap. Mean defect size was 180 ± 128 cm^2^, mean flap size was 278 ± 147 cm^2^, and mean operative time was 431 ± 109 min. Prior to free flap surgery, hypercoagulability due to hFVL mutation had already been confirmed in 14 flaps transferred on 11 patients (61%, Group A). The respective diagnoses had been established after the occurrence of deep vein thromboses (n = 4), fulminant pulmonary artery embolisms (n = 6) or miscarriages (n = 1). Consequently, these patients were already taking novel oral anticoagulants at the time of referral to our department. Table 1 summarizes the patients’ characteristics. Table 2 gives a patient- and flap-wise overview over the associated hypercoagulable conditions and additional risk factors.

**Table 1:** Patient characteristics and distribution of comorbidities

Patients	Total n (%)
Number of patients	15
Mean age [years] ± SD	54 ± 12
Median ASA class	2
Gender distribution [♀/♂]	5/10
Mean length of hospital stay [d] ± SD (range)	53 ± 76 (range: 8 to 274)
Mean postoperative length of hospital stay [d] ± SD (range)	51 ± 64 (range: 8 to 274)
Distribution of comorbidities	
Arterial hypertension (HTN)	7 (47%)
Coronary Artery Disease (CAD)	2 (13%)
Peripheral Artery Disease (PAD)	2 (13%)
Chronic Obstructive Pulmonary Disease (COPD)	6 (40%)
Chronic kidney disease (CKD)	1 (7%)
Diabetes Mellitus (DM)	4 (27%)
Obesity (Body Mass Index ≥ 30 kg/m^2^)	5 (33%)
Mean Body Mass Index (kg/m^2^)	29 ± 5

*ASA* American Society of Anesthesiologists, *cm* centimeter, *cm*^*2*^ square centimeter, kg kilogram, *n* numbers, *min* minutes, *SD* standard deviation

**Table 2:** Hypercoagulable states and additional risk factors

Flap#	Patient#	Type	Location	Anamnesis	Risk factors	Hypercoagulability
1A	1.1	DIEP right	Trunk	PAE	CRT	hFVL
2A	1.2	DIEP left	Trunk	PAE	CRT	hFVL
3A	2.1	DIEP	Trunk	DVT	Hormone therapy	hFVL, hPT, F VIII ↑
4A	3.1	LD/PSC	UE	DVT	–	hFVL, hPT
5A	4.1	VRAM	LE	DVT	Smoking	hFVL, hPT, F VIII ↑, Protein C/S ↓
6A	5.1	DIEP	Trunk	DVT	CRT	hFVL
7A	6.1	ALT	LE	PAE	–	hFVL, F VIII ↓, Protein S ↓
8A	7.1	Gracilis	LE	PAE	Smoking	hFVL, hPT, F VIII ↑, Lupusinhibitor
9A	7.2	LD	LE	PAE	Smoking	hFVL, hPT, F VIII ↑, Lupusinhibitor
10A	7.3	VRAM	LE	PAE	Smoking	hFVL, hPT, F VIII ↑, Lupusinhibitor
11A	8.1	DIEP	Trunk	Miscarriage	CRT	hFVL, Protein C ↓
12A	9.3	LD	LE	–	Smoking	hFVL, hPT, F VIII ↑, F IX ↑, F XI ↑
13A	14.2	LD	LE	–	–	hFVL, hPT, F VIII ↑,
14A	15.2	LD	LE	–	-	hFVL, hPT, F VIII ↑,
1B	9.1	LD	LE	–	Smoking	hFVL, hPT, F VIII ↑, F IX ↑, F XI ↑
2B	9.2	Gracilis	LE	–	Smoking	hFVL, hPT, F VIII ↑, F IX ↑, F XI ↑
3B	10.1	LD	LE	–	–	hFVL, hPT
4B	11.1	ALT	LE	–	Smoking	hFVL, hPT
5B	12.1	PSC	LE	–	Smoking	hFVL, hPT
6B	13.1	DIEP right	Trunk	–	CRT/Smoking	hFVL, F VIII ↓, Protein S ↓
7B	13.2	DIEP left	Trunk	–	CRT/Smoking	hFVL, F VIII ↓, Protein S ↓
8B	14.1	ALT	LE	–	–	hFVL, hPT, F VIII ↑,
9B	15.1	LD	LE	–	Smoking	hFVL, hPT, F VIII ↑,

A = Group A: hFVL diagnosis prior to free flap surgery

B = Group B: hFVL diagnosis after free flap surgery

*ALT* Anterior lateral thigh flap, *CRT* Chemoradiotherapy, *DIEP* Deep Inferior Epigastric Flap, *DVT* deep vein thrombosis, *F* female, *hFVL* heterozygotic factor V Leiden, *hPT* heterozygotic prothrombin mutation, *PAE* pulmonary artery embolism, *PSC* parascapular flap; VRAM = Vertical Rectus Abdominis Muscle

### Primary and secondary endpoints in Group A (n = 14 flaps)

Following preoperatively known diagnoses of hypercoagulability due to hFVL mutation (n = 14; 61%), one intraoperative arterial thrombosis occurred (7%). The flap was salvaged by means of immediate thrombectomy and re-anastomosis. Postoperative thromboses occurred in two flaps (14%). The first flap was lost due to fulminant flap pedicle thrombosis at the 1st postoperative day and repeated free flap surgery was required (7%). The second flap developed venous pedicle thrombosis at the 1st day post-surgery but was salvaged. The thrombotic vein was resected and reconstructed with an interpositional cephalic vein graft. Four flaps required re-explorations due to postoperative bleedings (n = 2, 14%) or pedicle compression due to hematoma formation or tissue swelling (n = 1, 7%, respectively). All flaps were salvaged. One flap, however, required further debridement and skin-grafting (n = 1, 7%).

### Primary and secondary endpoints in Group B (n = 9 flaps)

By contrast, in the cohort of flaps without preoperative confirmation of hFVL mutation, the incidence of both flap losses (primary endpoint) and major complications (secondary endpoint) were significantly higher. One flap showed an intraoperative arterial thrombosis after flap anastomosis but was salvaged by means of immediate thrombectomy and re-anastomosis (n = 1, 11%). Fulminant postoperative flap pedicle thromboses were seen in a total of 8 flaps (89%). In detail, three arterial thromboses (33%), three venous thromboses (33%) and two combined venous/arterial thromboses (22%). Three of these flaps could be salvaged, one by means of immediate thrombectomy and re-anastomosis, two with resection of the thrombotic arterial segment and reconstruction via an interpositional greater saphenous vein graft. Five flaps, however, developed substantial necroses, including four total losses, which led to reconstructive failures, and repeated free flap surgery was successfully carried out. Two flaps required additional debridement and skin-grafting. Table 3 shows flap- and patient-specific operative details and perioperative anticoagulation treatments**.**

**Table 3:** Operative details and anticoagulation treatment during and after flap surgery

Flap#	Patient#	Type	Arterial	Venous	Intraop. anticoagulation	Postop. anticoagulation
1A	1.1	DIEP	IMA	1 × CV	500 IU	15,000 IU/24 h
2A	1.2	DIEP	IMA	1 × CV	500 IU	15,000 IU/24 h
3A	2.1	DIEP	IMA	1 × CV	500 IU	15,000 IU/24 h
4A	3.1	LD/PSC	AR	1 × CV	1000 IU	2 × 0.6 Enoxaparin
5A	4.1	VRAM	PA via GSV	1 × CV	500 IU Bolus/300 IU/h	20,000 IU/24 h/ASS 100 mg
6A	5.1	DIEP	IMA	1 × CV	500 IU Bolus/300 IU/h	2 × 0.4 Enoxaparin
7A	6.1	ALT	PTA	1 × GSV	500 IU	15,000 IU/24 h
8A	7.1	Gracilis	PTA	2 × CV	500 IU	2 × 0.3 Enoxaparin
9A	7.2	LD	PTA	1 × CV	500 IU	2 × 0.6 Enoxaparin
10A	7.3	VRAM	Two stage—AVL		500 IU Bolus/300 IU/h	25,000 IU/24 h
11A	8.1	DIEP	IMA	1 × CV	500 IU	20,000 IU/24 h
12A	9.3	LD	PTA	1 × CV	2000 IU	20,000 IU/24 h
13A	14.2	LD	ATA	1 × CV	500 IU	2 × 0.3 Enoxaparin
14A	15.2	LD	ATA	1 × CV	1000 IU	25,000 IU/24 h/100 mg ASS
1B	9.1	LD	One stage—AVL		1000 IU	20,000 IU/24 h/Arixtra 2.5 mg
2B	9.2	Gracilis	PTA	1 × CV	500 IU	2 × 0.3 Enoxaparin
3B	10.1	LD	PTA	2 × CV	500 IU Bolus/300 IU/h/500 mg ASS	15,000 IU/24 h
4B	11.1	ALT	PTA	1 × GSV/1 × CV	500 IU	2 × 0.4 Enoxaparin
5B	12.1	PSC	Two stage—AVL		1000 IU	2 × 0.4 Enoxaparin
6B	13.1	DIEP	IMA	1 × CV	500 IU	2 × 0.3 Enoxaparin
7B	13.2	DIEP	IMA	1 × CV	500 IU	2 × 0.3 Enoxaparin
8B	14.1	ALT	ATA	1 × CV	500 IU	15,000 IU/24 h
9B	15.1	LD	PTA via GSV	1 × CV	500 IU	15,000 IU/24 h

A = Group A: hFVL diagnosis prior to free flap surgery

B = Group B: hFVL diagnosis after free flap surgery

*ALT* anterior lateral thigh flap, *ASS* Aspirin^®^, *ATA* Anterior Tibial Artery, *AVL* Arterio-Venous Loop, *CV* concomitant vein, *DIEP* Deep Inferior Epigastric Flap, *GSV* greater saphenous vein, *h* hour, *IMA* internal mammary artery, *IU* international unit, *LD* latissimus dorsi flap, *LE* lower extremity, *mg* milligram, *PA* popliteal artery, *PSC* parascapular flap, *PTA* posterior tibial artery, *UE* upper extremity, *VRAM* Vertical Rectus Abdominis Muscle Flap

### Comparison of flap loss incidences and major complications between groups

In summary, the overall rate of intraoperative flap thromboses was 9% (n = 2), whereas the overall rate of postoperative flap thromboses was 43% (n = 10). The corresponding salvage rates were 100% (n = 2/2) for intraoperative- and 40% (n = 4/10) for postoperative flap pedicle thromboses. Table 4 summarizes the distribution of flap losses and surgical complications between both groups. Upon comparing the cohort of flaps with hypercoagulable states known prior to free flap surgery and treated in accordance with our anticoagulation regimen (Group A, n = 14) versus those with hypercoagulability confirmed only after free flap surgery (Group B, n = 9), free flaps with an unconfirmed preoperative diagnosis had a 10 times higher risk for developing flap necroses (Group B = 4/9 versus Group A = 1/14; OR: 10.4; 95% CI 1.0, 134.7; p = 0.03).

**Table 4:** Groupwise comparison of surgical complications (n = number)

Intraoperative microvascular flap complications	Total (n %) n = 23	Group A (n %) n = 14	Group B (n %) n = 9
Arterial thrombosis	2 (9%)	1 (7%)	1 (11%)
Venous thrombosis	–	–	–
Venous/Arterial thrombosis	–	–	–
Total rate of intraoperative microvascular complications	2 (9%)	1 (7%)	1 (11%)
Postoperative microvascular flap complications			
Arterial thrombosis	3 (13%)	–	3 (33%)
Venous thrombosis	4 (17%)	1 (7%)	3 (33%)
Venous/Arterial thrombosis	3 (13%)	1 (7%)	2 (22%)
Total rate of postoperative microvascular complications	10 (43%)	2 (14%)	8 (89%)
Postoperative flap complications			
Total flap necrosis	5 (22%)	1 (7%)	4 (44%)
Partial flap necrosis	1 (4%)	–	1 (11%)
Wound healing disorder	3 (13%)	1 (7%)	2 (22%)
Hematoma	1 (4%)	1 (7%)	–
Bleeding	2 (9%)	2 (14%)	–
Total rate of major complications	12 (52%)	5 (36%)	7 (78%)
Donor-site complications			
Hematoma	2 (9%)	2 (14%)	–
Wound healing disorder	2 (9%)	–	2 (22%)
Infection	–	–	–
Seroma	–	–	–
Total rate of donor-site complications	4 (17%)	2 (14%)	2 (22%)

## Discussion

To date, there exists not enough information on the risks of microvascular thrombosis and flap failure in patients with hereditary thrombophilia [4]. This is especially relevant as hypercoagulable disorders are relatively common in the general population [14, 15]. In this present study we aimed at investigating one of the most frequent hereditary thrombophilias in the European population [7]. Currently there are very few studies investigating free flaps surgery in the context of FVL [3, 16, 17]. We evaluated our experience over the past decade, presenting the largest single-center cohort of patients with heterozygous FVL mutation undergoing free flap reconstructions. The goal of this study was to analyze outcome variables, complication rates, and anticoagulation regimens for these high-risk patients with an emphasis on the potential prognostic role of perioperative work-up. Furthermore, we sought to lay out our algorithmic approach for the preoperative risk assessment and anticoagulation therapy in this context. Thus, we hope to help increase patient safety and improve surgical outcomes in these thrombophilic patients.

Our findings show that in this high-risk patient cohort, the occurrence of flap thromboses can be significantly reduced and satisfactory flap outcomes can be achieved by adhering to strict perioperative risk assessment strategies and anticoagulation approaches. Nevertheless, it remains beyond doubt that these patients are at greater risk of reconstructive failure. Yet, our data suggests that preoperative diagnosis of hypercoagulability is associated with a significantly higher success rate in this context. In line with this, Friedman and colleagues concluded that preoperative vigilance on the part of the surgeon is important to identify patients with undiagnosed hereditary thrombophilia to decrease the incidence of thrombotic events [18]. Sezgin et al. highlighted the importance of preoperative screening questionnaires and showed that 21 patients (35%) had a positive history of hypercoagulable tendencies and reported that 9 patients (15%) were diagnosed with congenital thrombophilia after preoperative screening in their study [19]. In accordance with these findings, our results underline why the identification of hypercoagulable patients and the modification of anticoagulation regimens is crucial to prevent free flap failure. In our opinion, each patient needs to be evaluated by a standardized preoperative questionnaire based on family history, sex, age, comorbidities, and the cause and localization of their defect to prevent catastrophic outcomes. Furthermore, smoking, diabetes, peripheral vascular disease, delayed reconstruction, and older age are known risk factors that may lead to free flap failure [20, 21]. To complete preoperative diagnostics, blood- and coagulation analyses, including preoperative fibrinogen-, thrombocyte- and hemoglobin-levels, aPTT, Antithrombin III activity (AT III), should be performed routinely. As laid out in our suggested preoperative approach (Fig. 1), we recommend further diagnostics, such as a thrombophilia screening, whenever patient history or routine tests hint at abnormalities. However, it should be emphasized that the high success rate presented in this study might not only be due to meticulous preoperative diagnostics and consistent coagulation management. Other factors such as careful postoperative flap monitoring at an intermediate care unit, an aggressive revision policy, the right choice of flaps as well as the operations being performed by experienced microsurgeons might have contributed to this success.

Wang and colleagues presented a study including 41 patients with a history of a thrombotic event prior to surgery or a hypercoagulable state and reported an occurrence of thrombosis in 21% of their patients, which was lower than our results of postoperative flap pedicle thromboses (43%) [3]. In accordance with our results, they also observed an early occurrence of vascular complication in the first 2 days. In comparison to our salvage rate of 50% for postoperative thromboses, they reported a 100% flap failure rate if vascular compromise occurred at postoperative days 4 and 5, emphasizing the thrombogenic nature of these flaps. We agree with their conclusion, that when intraoperative thrombosis occurs, flap revascularization is feasible. However, if thrombosis occurs postoperatively, the risk for flap failure is much higher as compared to the general population [3]. For this reason our higher salvage rates might have also been achieved through an aggressive and fast revision policy, which might have been accomplished by careful postoperative flap monitoring.

APC-R phenotype and FVL mutation are prone to the occurrence of especially venous thrombosis [10]. Beneficial effects of LMW heparin over UFH are a lower risk of postoperative bleeding and fewer side effects such as heparin-induced thrombocythemia accompanied with a sufficient bioavailability [22]. Nevertheless, LMW heparin monitoring is limited by partial thromboplastin time and anti-factor Xa level adjustment [22]. Advantages of UFH are that it directly enters the bloodstream and quickly prevents clot formation. We therefore prefer UFH after the occurrence of intra- or postoperative thrombosis. In addition, due to its short bioactivity and reversibility, UFH is easier to monitor than LMW heparin. For the latter, frequent aPTT controls are obligatory to ensure the correct dosage.

Although our study provides important new insights into free flap surgery in high-risk patients with hFVL, our results have to be critically evaluated in the light of our study’s limitations. Primarily, our study is limited by its retrospective monocentric design, making it prone to a biased selection of cases and controls. In particular, patients with undiagnosed hypercoagulability are likely to have been missed during the retrospective charts review. In addition, the study cohort included only a relatively small sample size, without random patient allocation to either group. Therefore, only associations can be suggested, and real causalities cannot be demonstrated. Furthermore, it is important to point out that our study is limited by the heterogeneous group of patients with different thrombotic histories and additional co-mutations. Also, free flap surgeries were performed by multiple surgeons with different experience levels over a large time period. Flap losses and complications might have been due to technical errors, critical indications, and strategic mistakes, therefore. In addition, the patient cohort comprised cases of tumor and trauma reconstructions of the breast and the extremities. In this context, tumor patients were not excluded from our study, although it is known that they are at higher risk of hypercoagulability [23–25]. Finally, it has to be acknowledged that the pretest probability of the extended genetic work-up alone is relatively small, as the incidence of the mutation itself is relatively low. Therefore, to date, we advise against the prophylactic genetic screening of all microsurgical patients. Yet, meticulous pretest patient assessment with our anamnestic questionnaire helped identify over 60% of hFVL thrombophilia cases in our cohort. Amongst these, extended hypercoagulability work-up and subsequent adherence to our protocol was associated with a tenfold reduction in major complications.

In summary, our study represents the largest case–control series of free flaps in hypercoagulable patients with hFVL, underlining the importance of extensive preoperative work-up in suspected cases of thrombophilia.

## Conclusion

Patients with hFVL are at higher risk for free flap complications but successful reconstructions are feasible in cases with appropriate preoperative work-up and perioperative treatment-regimen. In this context, meticulous preoperative assessment is mandatory to detect patients with hypercoagulable disorders. To improve surgical results, a standardized preoperative diagnostic algorithm and subsequent anticoagulatory regimen for patients with hFVL must therefore be implemented and strictly adhered to. The suggested protocol can help identify patients with hFVL preoperatively, functioning as a clinical screening tool, considerably increasing the pretest probability of extended coagulation testing, thus contributing to patient safety in a cost-effective manner.

## Data Availability

The datasets used and/or analysed during the current study are available from the corresponding author on reasonable request.
